# Oral microbiota alterations in radiographic axial spondyloarthritis

**DOI:** 10.3389/fmed.2026.1815404

**Published:** 2026-05-12

**Authors:** Aigerim Abuova, Assel Meiramova, Yekaterina Zueva, Jeannette Kunz, Laura Chulenbayeva, Argul Issilbayeva, Zharkyn Jarmukhanov, Elizaveta Vinogradova, Bayan Ainabekova, Samat Kozhakhmetov, Almagul Kushugulova

**Affiliations:** 1 Department of Internal Medicine #2, Astana Medical University, Astana, Kazakhstan; 2 Scientific Department, Art Science Innovation Center, Astana, Kazakhstan; 3 Department of Molecular Biology, Medical Faculty, Ariel University, Ashkelon, Israel; 4 Department of Biomedical Sciences, School of Medicine, Nazarbayev University, Astana, Kazakhstan; 5 Laboratory of Microbiome, Center for Life Sciences, National Laboratory Astana, Nazarbayev University, Astana, Kazakhstan

**Keywords:** *Actinomyces*, oral microbiome, *Porphyromonas*, radiographic axial spondyloarthritis, *Saccharimonadaceae*, Th17 inflammation

## Abstract

**Objective:**

To investigate alterations in the oral microbiome of patients with radiographic axial spondyloarthritis (r-axSpA), to identify microbial taxa associated with disease status and structural progression, and to explore potential links between oral microbiota composition and systemic immunological profiles.

**Methods:**

An observational cross-sectional study was conducted including 57 radiographic axial spondyloarthritis patients and 41 healthy controls. Oral samples were analyzed using 16S rRNA gene sequencing; amplicon sequence variants (ASVs) were generated using LotuS2/DADA2 and taxonomically annotated with SILVA, Greengenes, and HITdb. Alpha and beta diversity were assessed using ACE, Pielou indexes, and UniFrac distances with PERMANOVA and ANOSIM. Differential abundance was determined via LEfSe (LDA > 2, *p* < 0.05). Associations between microbial taxa, disease activity, structural damage, and immunological markers were evaluated using linear modeling and Spearman correlation analysis.

**Results:**

R-axSpA patients showed alterations in oral microbiome composition compared with controls, although alpha diversity remained largely comparable. Actinobacteria, Spirochaetes, and Synergistetes tended to be enriched in r-axSpA patients, mainly driven by an increased abundance of *Actinomyces* and Selenomonas. Several key periodontal pathogens, including *Porphyromonas gingivalis* and *Actinomyces* species, were more abundant in the r-axSpA group. Within the r-axSpA group, the abundance of *Porphyromonas* and *Saccharimonadaceae* showed associations with the severity of sacroiliitis and ankylosis. These taxa also exhibited positive correlations with systemic pro-inflammatory cytokines (such as IL-17), which may suggest a possible link between oral dysbiosis and enhanced Th17-driven inflammation in r-axSpA.

**Conclusion:**

Patients may exhibit a distinct proinflammatory oral microbiome profile, with increased representation of *Actinomyces* and *Porphyromonas* species. Certain microbial taxa, including members of Porphyromonadaceae and Patescibacteria, have been reported to correlate with cytokines implicated in the immunopathogenesis of radiographic axial spondyloarthritis. These observations suggest that oral dysbiosis could play a role in the maintenance or modulation of systemic inflammation in r-axSpA, and that the oral microbiome might serve as a potential source of biomarkers or a target for future therapeutic strategies.

## 1 Introduction

Radiographic axial spondyloarthritis (r-axSpA) is a chronic inflammatory rheumatic disease. It primarily affects the axial skeleton, including entheses and peripheral joints and occasionally, it involves extra-articular organs and systems (1). The disease has a prevalence of 2–37 cases per 10,000 individuals across diverse racial and geographic populations (2–4). In Kazakhstan, according to official statistics, the prevalence of r-axSpA is 2 per 10,000 population (5). R-axSpA predominantly affects males and typically manifests before age 45. This often leads to significant disability and reduced quality of life during peak productive years (6–9).

The etiology of r-axSpA remains incompletely understood, with ongoing research focused on its multifactorial pathogenesis involving genetic predisposition (notably the HLA-B27 and ERAP1 gene variants), immune dysregulation (IL-23/IL-17 and NF-kβ pathways activation), and microbial dysbiosis (10).

The role of the microbiome is well-researched in rheumatoid arthritis (RA) (11, 12). Gut dysbiosis can precede the clinical onset of RA (13) and is associated with disease severity (14). Oral microbiome imbalances are also linked to RA pathogenesis (15–17). Similarly, gut dysbiosis is frequently observed in AS (18, 19) and is associated with worse disease activity and impaired physical function (20); however, far fewer studies are available on AS compared to RA. Dysbiosis in r-axSpA can promote inflammation through various mechanisms, including activation of the IL-23/IL-17 pathway (10), which can drive inflammation in both the gut and joints. Studies have consistently shown that AS patients exhibit an altered gut microbial composition compared to healthy individuals, characterized by reduced overall diversity and shifts in the abundance of specific bacterial families (18). Relatively consistent findings include an increase in Prevotellaceae and Enterobacteriaceae, and less consistent increases in Bacteroidaceae, Actinomycetaceae, and Porphyromonadaceae (18). Furthermore, impairment of the gut vascular barrier has been documented in AS, accompanied by significant upregulation of zonulin and associated with elevated serum levels of LPS (21).

A close association between periodontal disease (PD) and axial spondyloarthritis (axSpA) has long been speculated (22–24); however, surprisingly few studies have investigated oral microbiome changes in axSpA. The limited available studies consistently indicate alterations in alpha diversity compared to healthy controls, though the direction and magnitude of these changes vary (25–27). Furthermore, these studies report diverse microbial markers. For instance, Bisanz et al. found no strong evidence of specific taxa associated with axial axSpA in subgingival plaque (25) but observed strong negative associations between *Actinomyces* spp., *Streptococcus* spp., and oral health in axSpA patients as measured by probing pocket depth (PPD). In contrast, Stoll et al. highlighted increased abundance of Fusobacterium in plaque and *Rothia mucilaginosa* in saliva as key markers in juvenile r-axSpA (26). Gill et al. identified enrichment of Prevotellaceae, Actinobacillus, Corynebacterium, Treponema, and several other taxa in the salivary microbiome of axSpA patients (28). Lv et al. reported depletion of Streptococcus and enrichment of Veillonellaceae, as well as opportunistic pathogens including *Brucella* spp. and *Campylobacter concisus*, in AS saliva (27). Separately, Öğrendik M. observed that anti-*P. gingivalis* and anti-*Prevotella intermedia* antibody titers are significantly higher in patients with AS than in healthy controls (29).

It is hypothesized that alterations in the human microbiome could contribute to the pathogenesis of HLA-B27-associated axSpa. This observation aligns with HLA-B27 transgenic and human-to-mouse FMT models, where HLA-B27 expression alters the gut microbiome and promotes disease: arthritis and inflammation do not develop in germ-free conditions, but readily appear upon colonization with axSpa-associated microbiota from HLA-B27-positive donors (30). These models demonstrate that bacteria are necessary to trigger disease, potentially through mechanisms like molecular mimicry (e.g., homology between bacterial antigens and HLA-B27 peptides) (31–33).

Studies have reported that macrophages from AS patients exhibit higher expression of IL-23, a key pro-inflammatory cytokine, in response to lipopolysaccharide (LPS) (10). IL-23/IL-17 axis drives chronic inflammation in diseases like periodontitis, where microbial products trigger epithelial cells to produce IL-23, which, in turn, promotes Th17 cell differentiation and IL-17 production, leading to systemic effects that can exacerbate other inflammatory conditions. In particular, in the oral cavity, pathobionts such as *Porphyromonas gingivalis* can stimulate IL-23 production, a key driver of autoimmune diseases, primarily through its LPS. This mechanism, particularly under dysbiotic conditions, could potentially exacerbate axial inflammation in AS (34, 35).

The oral microbiome is increasingly recognized as a significant contributor to systemic diseases, a perspective substantiated by research in other conditions, as outlined in the reviews by Baker et al. (36) and Rajasekaran et al. (35). For instance, *Porphyromonas gingivalis* is implicated in RA through the induction of production of anti-citrullinated protein antibodies (ACPA) (37, 38) and has also been linked to neuroinflammation and amyloid plaque accumulation in Alzheimer’s disease (39). Similarly, *Fusobacterium nucleatum* was found to be associated with colorectal carcinogenesis through modulation of E-cadherin/β-catenin signaling, promoting cell proliferation (40).

Research indicates that oral bacteria can promote systemic inflammation through various mechanisms, including immune dysregulation, gut barrier disruption, ectopic colonization, and molecular mimicry. Given the emerging evidence implicating the microbiome in the pathogenesis of autoimmune diseases, and in r-axSpa in particular, investigating the mechanisms that may be operative in r-axSpa is a pressing need. The current gap in understanding the oral microbiome’s role in r-axSpa underscores the demand for further research. Our study aimed to address this gap by comparing the diversity and composition of the oral microbiome between r-axSpA patients and healthy controls, thereby paving the way for new insights into the disease. To our knowledge, this is the first study of its kind conducted in Kazakhstan and Central Asia.

## 2 Materials and methods

### 2.1 Participants and recruitment

**Figure 1:**
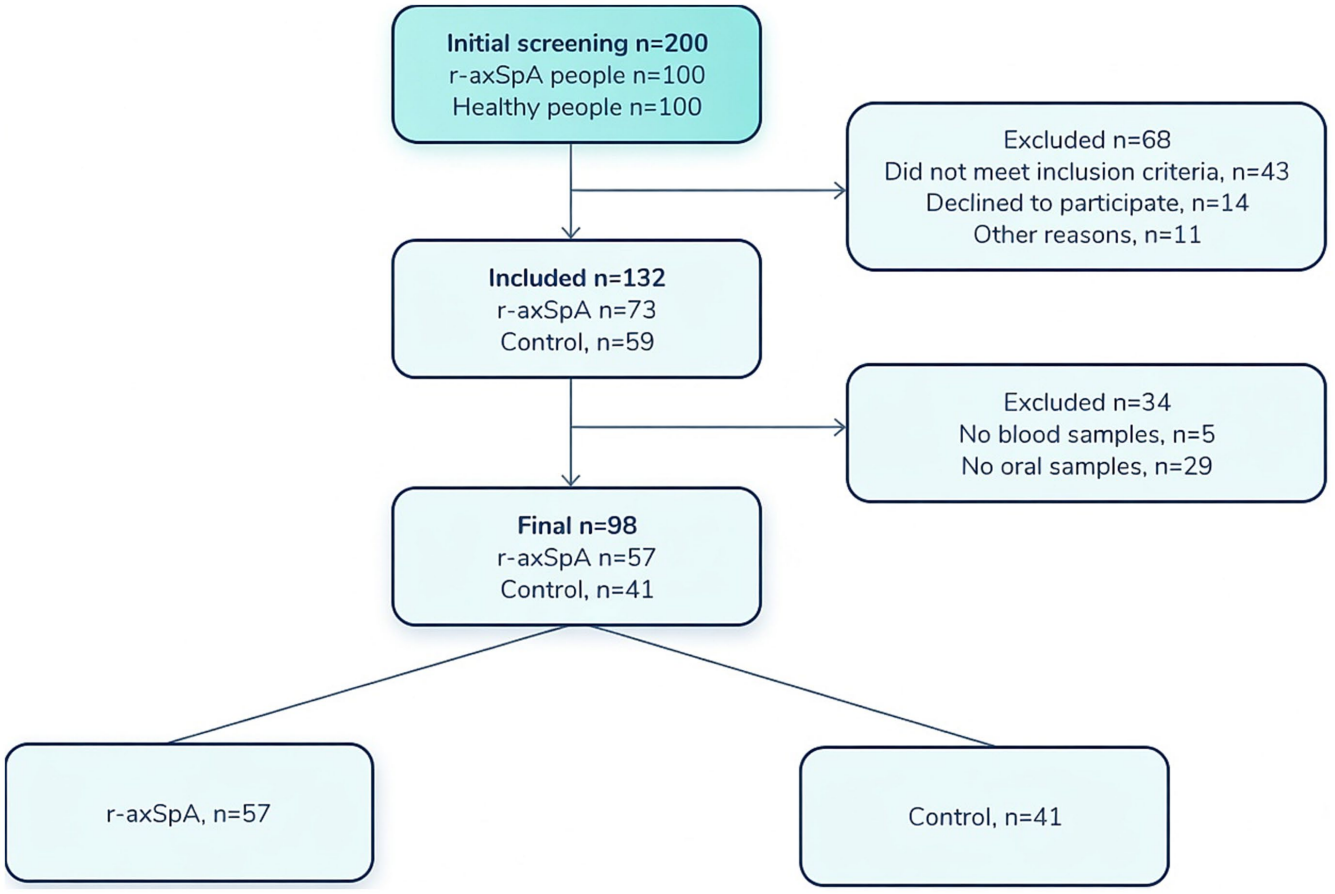
STARD diagram showing the flow of participants through the study.

We performed an observational analytical one-stage cross-sectional study at the City Multifunctional hospital #2 in Astana. The study period extended from 01.02.2024 to 25.12.2024, during which a total of 100 subjects with diagnosis of r-axSpA were enrolled. All the patients were examined by a rheumatologist to confirm a diagnosis of r-axSpA based on the modified (New York) criteria classification. Subjects were excluded if they were younger than age 18 years, pregnant or lactating, had a history of chronic somatic diseases, had an oral disease, detected during an oral examination, had an inflammatory bowel disease or had antibiotic\probiotics use 6 months prior to sample collection. Following the application of exclusion criteria, 73 participants were selected for inclusion in the main group of the study. During the study 3 patients refused to continue participating, 13 patients had a poor quality of oral microbiome samples, the main group consisted of 57 patients (Figure 1).

The control group of the study consisted of 41 healthy individuals who had no verified chronic diseases, who had not taken antibiotics or probiotics for the last 6 months.

All individuals included in the study, both from the main and control observation groups, passed a clinical examination. During the clinical examination, complaints, medical and life history, and objective examination were collected. Anamnesis was conducted with the identification of risk factors for the development and possible triggers of the disease. In addition, the following were noted: infectious diseases, traumas and surgical interventions and allergic aggravation. Data on bad habits and occupational hazards, housing and living conditions were recorded on individual cards. When collecting anamnesis of the disease, the following data were taken into account: disease debut, patient’s age at the time of r-axSpA debut, duration of the disease, subjective connection of the disease with some endogenous or exogenous trigger and period from the disease debut to the diagnosis of r-axSpA.

The bone and joint system was examined in accordance with generally accepted rules. The functional disorders of the spine and joints were assessed using BASFI (The Bath Ankylosing Spondylitis Functional Index) and BASMI (The Bath Ankylosing Spondylitis Metrology Index) indices. Disease activity was gauged using the ASDAS (Ankylosing Spondylitis Disease Activity Score). Data on extra-articular manifestations of the disease and the development of complications were recorded. Stages of ankylosis were diagnosed according to CT scans of the spine and ileosacral articulations of patients in the last year according to the Kellgren classification.

All patients underwent laboratory blood examination in the clinical diagnostic laboratory. Blood sampling was performed strictly on an empty stomach, after a 12–14 h fasting period. General clinical examinations were performed, with determination of the biochemical examination including the assessment of erythrocyte sedimentation rate (ESR) and C-reactive protein (CRP) levels. Genetic testing for the presence of HLA B27 was performed.

### 2.2 Evaluation of periodontal health in patients with r-axSpA and control group

A dentist performed the clinical evaluation of the participants, which included a gathering of patient complaints and disease history, an inspection of the oral cavity, and an indexed evaluation of the periodontal tissues’ status. The questionnaire captured reports of gum bleeding—its duration, triggers, and circumstances—as well as the rate of halitosis, tooth looseness, esthetic concerns from shifting anterior teeth, heightened tooth sensitivity to diverse stimuli, and disruptions in the oral functional system. Diagnostic X-rays were taken as required for confirmation. Periodontal health was characterized by probing depths of ≤3 mm across sites without bleeding on probing. Periodontitis was identified when probing depths reached ≥4 mm, with ≥2 mm clinical attachment loss and <15% radiographic bone loss.

### 2.3 Oral sample collection, DNA extraction and sequencing

All patients included in the study had their oral microbiome samples collected. Prior to material collection, all study participants were advised to refrain from eating and hygienic procedures for a period of 2 h. Material was collected using a kit in accordance with the protocol of the manufacturer, ZYMO RESEARCH USA.

DNA extraction from the participants’ materials was performed at the National Laboratory Astana, Nazarbayev University, using kits. Genomic DNA from oral samples was extracted using the ZymoBIOMICS DNA Miniprep Kit (Zymo Research, D4300), and sterile μQ water was used as a negative extraction control. A qualitative control of DNA isolation was performed by OD260/280 Nanodrop and electrophoresis in a 1% agarose gel. The concentration and purity of each DNA sample were determined using an Invitrogen Qubit 3.0 Fluorimeter (Invitrogen, Carlsbad, CA, United States). Sterile μQ water served as a negative control. Sequencing was performed on the Illumina NovaSeq 6000 platform at the laboratory of Novogene (Beijing, China) following the standard Illumina protocols.

### 2.4 Processing of 16S rRNA amplicon sequencing data

Raw 16S rRNA reads were processed into amplicon sequence variant (ASV) abundance tables using LotuS2 v2.23. Demultiplexing, quality filtering, and dereplication were performed with the built-in simple demultiplexer using the MiSeq SDM configuration. A total of 19,589,012 reads were processed, of which 15,816,329 passed primary quality filters. ASVs were inferred with DADA2. Putative chimeras were removed *de novo* within the pipeline. Taxonomic assignment used LAMBDA with an LCA strategy against SILVA 138.1, Greengenes 13.5, and HITdb. After post-filtering, the final feature table contained 2,261 ASVs and 18,014,920 total reads.

### 2.5 Prediction of metabolic pathways

Functional profiling of microbial communities was carried out using PICRUSt2 (version 2.5.0) under default parameters. This approach estimated gene family copy numbers for each ASV by referencing a phylogenetic tree and applying a Nearest Sequenced Taxon Index (NSTI) cutoff of 2. Predicted metabolic pathway abundances were annotated using the MetaCyc Metabolic Pathway database.

### 2.6 Statistical analyses

Statistical analysis and visualization were performed in Python v3.12 using NumPy v2.0.1, SciPy v1.15.1, statsmodels v0.14.4, scikit-learn v1.6.1, Matplotlib v3.10.0, and seaborn v0.13.2 packages. Biodiversity analysis was performed using scikit-bio v0.6.3. Taxonomic data were rarified prior to analysis. The baseline characteristics of the groups were compared using Fisher’s exact test, independent t-test, with or without Welsh correction, or Mann–Whitney U rank test, where appropriate according to the data distribution parameters. Differential analysis was performed using LEfSe. For the differential analysis, markers were considered significant at *p* < 0.05 and LEfSe LDA > 2. Only features with a prevalence of at least 25% (in any group) were considered for analysis. Between-group diversity was assessed using unweighted Uni-Frac (U-UniFrac) and weighted Uni-Frac (W-UniFrac) distances. The significance of grouping was assessed using nonparametric ANOSIM and parametric PERMANOVA tests with 999 permutations. The PERMDISP test was performed before the PERMANOVA analysis to assess the equality of variances. Principal coordinate analysis (PCoA) was used to visualize compositional differences between samples. Within-sample diversity was assessed at the ASV level using the ACE index (a measure of richness) and the Pielou index (a measure of evenness). Correlation analysis between marker taxa, pathways, and clinical and immunological parameters was performed using Spearman’s coefficient without adjustment for multiple comparison.

## 3 Results

### 3.1 Study participants and their characteristics

A total of 98 subjects were enrolled in the study, including 57 patients with radiographic axial spondyloarthritis (r-axSpA) and 41 controls (Cntrl) (Table 1). A large proportion of the r-axSpA cohort (71.9%, *n* = 41) were receiving conventional disease-modifying antirheumatic drugs (cDMARDs) at the time of sampling. Among the 57 r-axSpA patients, 83% tested positive for HLA-B27, while 13% tested negative.

**Table 1:** Demographic and clinical characteristics of the studied groups.

Parameters	Control (*n* = 41), Cntrl	Patients (*n* = 57), r-axSpA	*p*-Value
Demographic characteristics			
Age, years, Md (IQR)	37.0 (29.0; 43.0)	40.0 (32.0; 49.0)	0.14^1^
Sex, F/M	3/38	12/45	0.09^2^
Age of r-axSpA onset, years, Md (IQR)	–	24.0 (19.0; 33.0)	
Disease duration, years, Md (IQR)	–	12.0 (5.0; 21.0)	
Hereditary of r-axSpA, %	–	11 (19.3%)	
Degree of sacroiliitis, 1/2/3/4, *n*	–	7/21/16/13	
Ankylosis, *n* (%)	–	29 (50.9%)	
Kyphosis, *n* (%)	–	24 (42.1%)	
Periodontal characteristics			
Oral health, *n* (%)	37 (90.2%)	50 (87.7%)	0.76^1^
PD mm, Md (IQR)	1.3 (0.4; 1.7)	3.8 (2; 6)	0.01^3^
CAL mm, Md (IQR)	1.7 (0.6; 2.2)	1.6 (1.1; 2.0)	0.44^3^
Tooth loss, n, Md (IQR)	4.1 (1.4; 5.3)	5.0 (2.1; 6.8)	0.16^3^
BoP, Md (IQR)	1.4 (0.5; 1.8)	1.8 (0.8; 2.4)	0.39^3^
PI, Md (IQR)	41.0 (13.9; 52.7)	45.1 (18.9; 60.9)	0.64^3^
Laboratory characteristics			
CRP, mg/L, Md (IQR)	–	4.3 (1.7; 14.2)	
HLA-B27 positive, *n* (%)	–	47 (82.5%)	
Therapy			
cDMARDs, *n* (%)		41 (71.9%)	
bDMARDs, *n* (%)		16 (20.1%)	
Disease activity			
ASDAS-CRP ≤ 2.1, score, *n* (%) (inactive r-axSpA)	–	24 (42.1%)	
ASDAS-CRP > 2.1, score, *n* (%) (active r-axSpA)	–	33 (57.9%)	
CRP ≤ 5.1, mg/L, *n* (%) (low inflammation)	–	30 (52.6%)	
CRP > 5.1, mg/L, *n* (%) (high inflammation)	–	*n* (47.4%)	

1 Ind. T-test.

2 Fisher’s exact test.

3 Mann-Whitney U rank test.

Md (IQR), Median (Interquantile range); r-axSpA, radiographic axial spondyloarthritis; HC, healthy control; PD, pocket depth; CAL, clinical attachment level; BoP, bleeding on probing; PI, plaque index; CRP, C-reactive protein; cDMARDs, conventional disease-modifying antirheumatic drugs; ASDAS-CRP, ankylosing spondylitis disease activity score with CRP.

### 3.2 Microbial profile of the oral cavity in r-axSpA group

**Figure 2:**
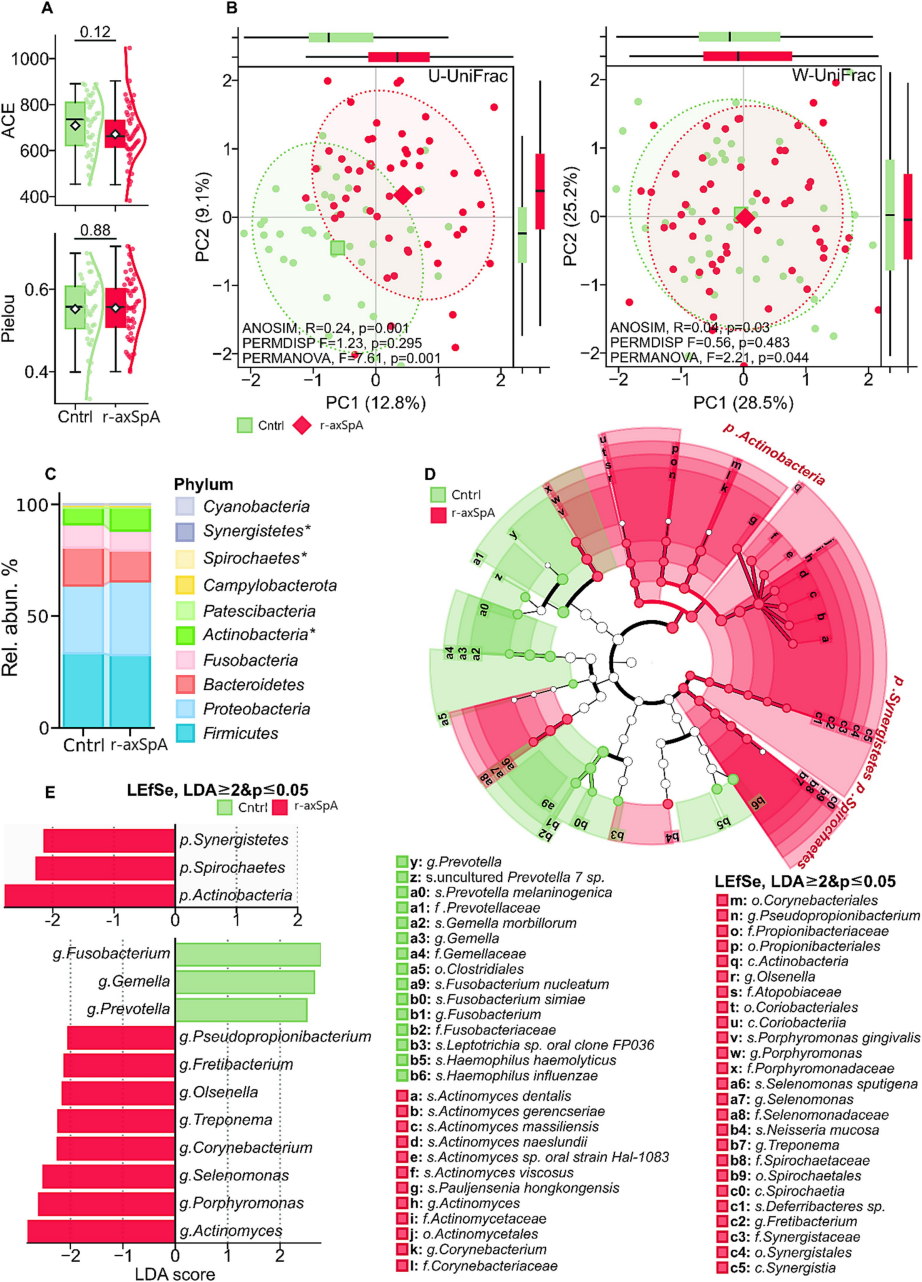
Variation in microbial diversity and relative taxa abundance in the oral microbiome of control (Cntrl) and radiographic axial spondyloarthritis (r-axSpA) groups. **(A)** Within-sample taxonomic diversity analysis (α-diversity). Boxplots with kernel density estimates (KDE) of distributions of taxonomic richness and evenness in Cntrl and r-axSpA groups. ACE and Pielou indexes. **(B)** Between-sample taxonomic diversity analysis (β-diversity). Scatter plot with 95% confidence intervals of principal coordinate analysis (PCoA) decomposition of taxonomic structure based on unweighted (U) and weighted (W) UniFrac distances at the ASV level in Cntrl and r-axSpA groups. ANOSIM test of significance of grouping with 999 permutations. **(C)** Bar chart showing therelative abundances of bacterial phyla fractions in Cntrl and r-axSpA groups. **(D)** Cladogram illustrating significant shifts in taxonomic abundance between Cntrl and r-axSpA groups across multiple phylogenetic levels. **(E)** Bar plot of LEfSe LDA scores for differentially abundant phyla and genera. ASV = amplicon sequence variant.

Alpha diversity estimates were largely similar between groups. Taxonomic richness, as measured by the ACE index, showed a non-significant decreasing trend in the r-axSpA group compared to the Cntrl group (*p* = 0.12). Taxonomic evenness, assessed using the Pielou index, was comparable between the groups (*p* = 0.88) (Figure 2A).

Beta diversity analyses indicated that microbial community structure differed significantly between the groups. Both unweighted (U) and weighted (W) UniFrac distance metrics demonstrated a significant group separation, as determined by PERMANOVA (U-UniFrac: *F* = 7.61, *p* = 0.001; W-UniFrac: *F* = 2.21, *p* = 0.044) and ANOSIM (U-UniFrac: *R* = 0.24, *p* = 0.001; W-UniFrac: *R* = 0.04, *p* = 0.03) tests (Figure 2B).

Differential analysis revealed significant shifts in the relative abundance of multiple taxa in the r-axSpA group (LEfSe, LDA ≥ 2, *p* ≤ 0.05). At the phylum level, the relative abundances of *Actinobacteria* (primarily driven by an increase in the family *Actinomycetaceae*, genus *Actinomyces*), *Spirochaetes*, and *Synergistetes* were significantly increased in r-axSpA patients compared to the Cntrl group (*p* = 0.004, *p* = 0.016, and *p* = 0.005, respectively). The prominent phylum *Actinobacteria* increased more than two-fold, with a median abundance of 4.67% (IQR: 2.92; 9.68) in Cntrl compared to 9.46% (IQR: 5.37; 14.27) in the r-axSpA group. The rarer taxa *Spirochaetes* increased from 0.04% (IQR: 0.01; 0.11) in Cntrl to 0.11% (IQR: 0.04; 0.26) in r-axSpA patients. Similarly, *Synergistetes* increased from a median of 0.002% (IQR: 0.0; 0.005) in Cntrl to 0.006% (IQR: 0.001; 0.038) in the r-axSpA group. A bar plot representing the structure of the oral microbiome at the phylum level is shown in Figure 2C.

At the family level (Figure 2D), the r-axSpA group was characterized by a significant increase in the relative abundance of multiple taxa, including *Actinomycetaceae*, *Porphyromonadaceae*, *Selenomonadaceae*, *Spirochaetaceae*, *Corynebacteriaceae*, *Synergistaceae*, *Atopobiaceae*, and *Propionibacteriaceae*. Specifically, the median abundance of *Actinomycetaceae* was 2.07% (IQR: 1.18; 3.77) in Cntrl compared to 3.65% (IQR: 2.15; 7.78) in the r-axSpA group. Similarly, *Porphyromonadaceae* increased from 0.27% (IQR: 0.07; 0.80) to 0.48% (IQR: 0.21; 1.35), and *Selenomonadaceae* from 0.15% (IQR: 0.05; 0.47) to 0.50% (IQR: 0.10; 0.78).

At the genus level (Figures 2D,E), the largest increases were observed in *Actinomyces*, *Porphyromonas*, *Selenomonas*, *Corynebacterium*, *Olsenella*, *Treponema*, *Fretibacterium*, and *Pseudopropionibacterium*. The relative abundance of *Actinomyces* increased from 1.57% (IQR: 0.89; 2.70) in Cntrl to 3.48% (IQR: 1.76; 6.86) in r-axSpA. Similarly, *Porphyromonas* increased from 0.22% (IQR: 0.07; 0.80) to 0.48% (IQR: 0.21; 1.35), and *Selenomonas* from 0.14% (IQR: 0.05–0.44) to 0.48% (IQR: 0.10–0.71).

At the species level, the largest differences in microbial community structure were driven by a significant increase in the relative abundance of *Porphyromonas gingivalis*, *Actinomyces viscosus*, *Actinomyces naeslundii*, *Actinomyces dentalis*, *Actinomyces gerencseriae*, *Actinomyces massiliensis*, *Actinomyces* sp. *oral strain Hal-1083*, *Pauljensenia hongkongensis*, *Deferribacteres* sp., and *Selenomonas sputigena*.

This redistribution of relative abundance in the r-axSpA group was characterized by a decrease in the relative fraction of *Prevotellaceae*, *Fusobacteriaceae*, *Gemellaceae*, and their corresponding species, as well as the species *Haemophilus haemolyticus* and *Haemophilus influenzae*. This conclusion is supported by their relative significant increase in the Cntrl group (Figure 2D).

The microbiome profiles of r-axSpA patients were predominantly characterized by a significant and consistent increase in the abundance of multiple members of the phylum *Actinobacteria*, as well as increases in the phyla *Spirochaetes* and *Synergistetes*, and taxa from the genus *Porphyromonas*. This highlights a shift in taxonomic structure associated with the disease state. Notably, the majority of these differential markers are associated with dental health issues and/or periodontal disease. Key species included *Porphyromonas gingivalis*, *Actinomyces viscosus*, *Actinomyces dentalis*, *Pauljensenia hongkongensis*, *Deferribacteres* sp., and *Selenomonas sputigena*.

### 3.3 Microbiome profiles in the r-axSpA group across disease progression and structural complications

We further examined differences in the taxonomic composition of individuals with r-axSpA based on structural complications, specifically investigating associations with sacroiliitis and ankylosis.

**Figure 3:**
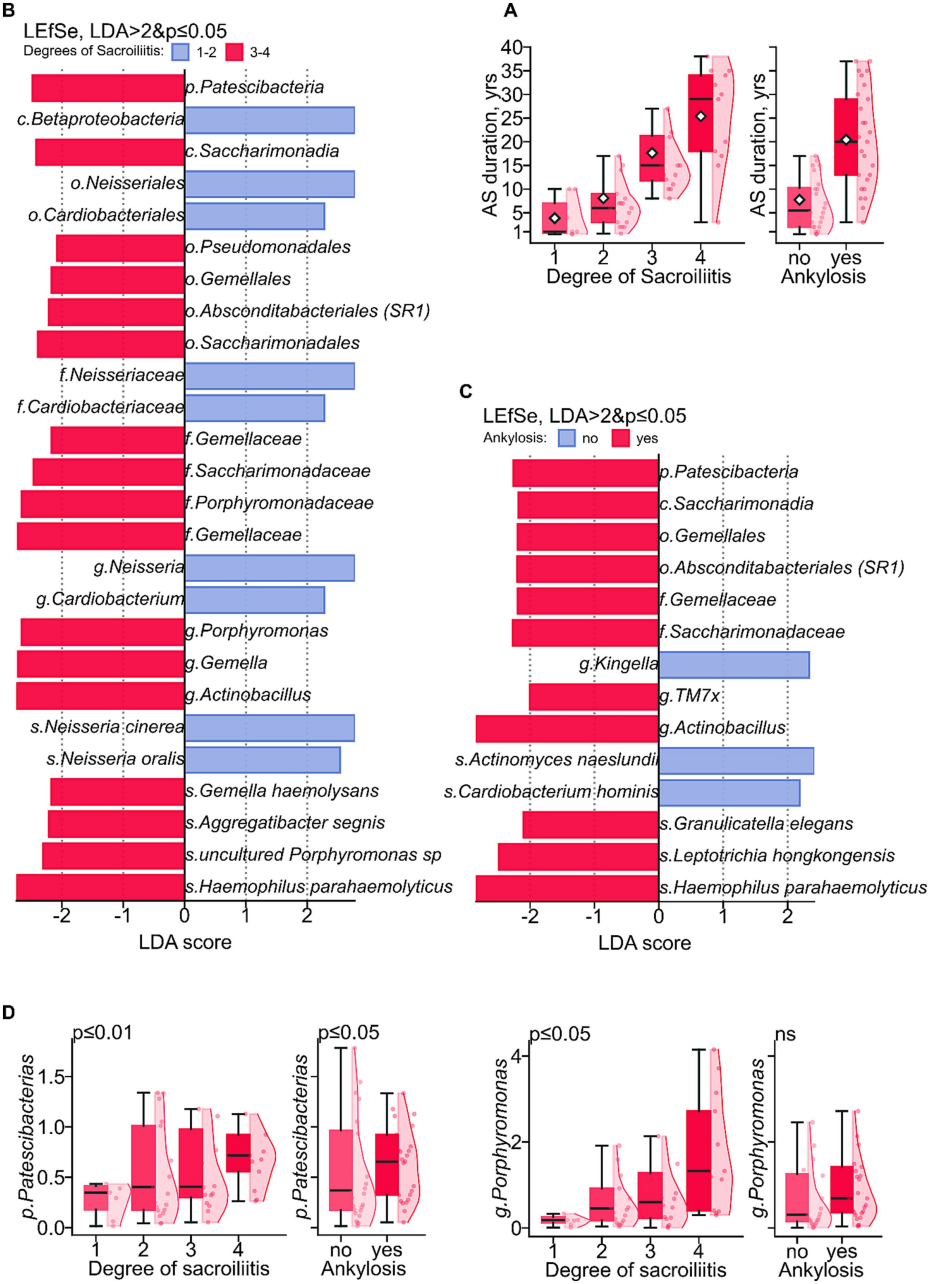
Dynamics of disease duration and complication development. **(A)** Boxplots with kernel density estimate (KDE) of disease duration distributions associated with sacroiliitis and ankylosis development. **(B)** Bar plot of LEfSe LDA scores for differentially abundant taxa associated with sacroiliitis degree. **(C)** With ankylosis development. **(D)** Boxplots with KDE of taxonomic marker relative abundance associated with sacroiliitis degree and ankylosis development at genera level.

Sacroiliitis, the initial inflammation of the sacroiliac joints, is often the first manifestation of r-axSpA. Over time, progressive stiffness and fusion of the joints (i.e., ankylosis) can occur, although the rate of progression varies greatly among individuals. We observed that the onset of these complications in our data corresponded with disease duration. Sacroiliitis progression occurred in increments corresponding to a median of 4.5-year intervals, while the onset of ankylosis manifested after a median of 14.5 years of disease duration (Figure 3A).

Differential analysis revealed a number of microbial markers associated with disease progression (Figures 3B,C). Specifically, the phylum *Patescibacteria,* family *Saccharimonadaceae* and the genera *Actinobacillus* were significantly associated with both sacroiliitis and ankylosis (Figure 3B). *Porphyromonas* was significantly associated only with sacroiliitis. Furthermore, the relative abundance of *Patescibacteria* and *Porphyromonas* taxa showed a significant linear association with the degree of sacroiliitis (Figure 3D).

### 3.4 Microbiome profiles in the r-axSpA group across gender and laboratory-clinical criteria

We further examined differences in the taxonomic composition of individuals with r-axSpA based on their laboratory-clinical criteria.

**Figure 4:**
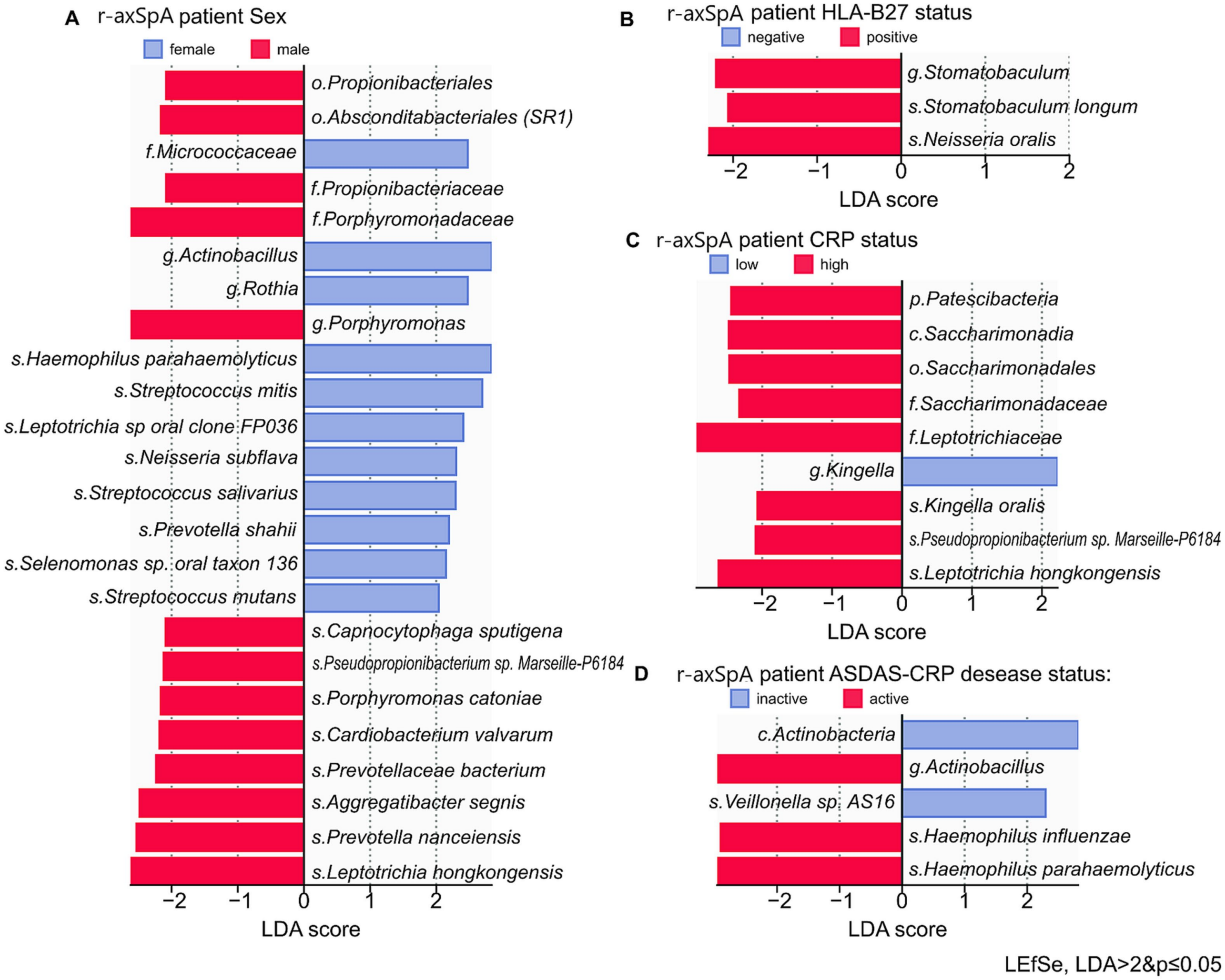
Differences in the taxonomic composition of the oral microbiome in individuals with r-axSpA, stratified by gender and laboratory-clinical criteria. **(A)** Bar plot of LEfSe LDA scores for differentially abundant taxa focusing on r-axSpA patients’ gender. **(B)** Focusing on HLA B27 status. **(C)** Focusing on CRP level. **(D)** Focusing on ASDAS-CRP disease status.

We examined the association between oral microbiome composition and patient sex in r-axSpA, revealing distinct microbial markers (Figure 4A). The oral microbiome of male r-axSpA patients (*n* = 45) was significantly enriched in members of the orders *Propionibacteriales* and *Absconditabacteriales (SR1)*, the families *Propionibacteriaceae* and *Porphyromonadaceae*, and the genus *Porphyromonas*. In contrast, female r-axSpA patients (*n* = 12) exhibited increased abundances of the genera *Rothia* and *Actinobacillus* (*p* < 0.05).

We further assessed the influence of HLA-B27 status on microbiome structure. In HLA-B27-positive r-axSpA patients (*n* = 37), a significant increase in the abundance of genera *Stomatobaculum*, species *Stomatobaculum longum* and *Neisseria oralis* was observed (*p* < 0.05) (Figure 4B). Subsequently, we investigated associations between oral microbiota and systemic inflammation, as measured by C-reactive protein (CRP) levels. High CRP levels in r-axSpA patients (*n* = 27) were associated with increased abundance of representatives of the phylum *Patescibacteria*, class *Saccharimonadia* (*p* < 0.05), whereas low CRP levels were associated with enrichment in the genus *Kingella* (Figure 4C). Considering the impact of disease activity, as reflected by the ASDAS-CRP status, we examined differences between patients with active disease (*n* = 36) and those with inactive disease (*n* = 21). Active disease was associated with elevated levels of the genus *Actinobacillus* (*p* < 0.05), while inactive disease was characterized by a relative enrichment in the species *Velonella* sp. *AS16* (Figure 4D).

Overall, the majority of subgroup differences were associated with patients’ gender, potentially reflecting the known male predisposition to r-axSpA. Stratification by other laboratory-clinical criteria did not yield strong or coherent microbial signatures, suggesting an overall homogeneity of the r-axSpA oral microbiome, with a consistent increase in r-axSpA-associated markers, such as the genus *Actinomyces*, across all subgroups.

### 3.5 Functional profiles of the oral microbiome associated with r-axSpA

**Figure 5:**
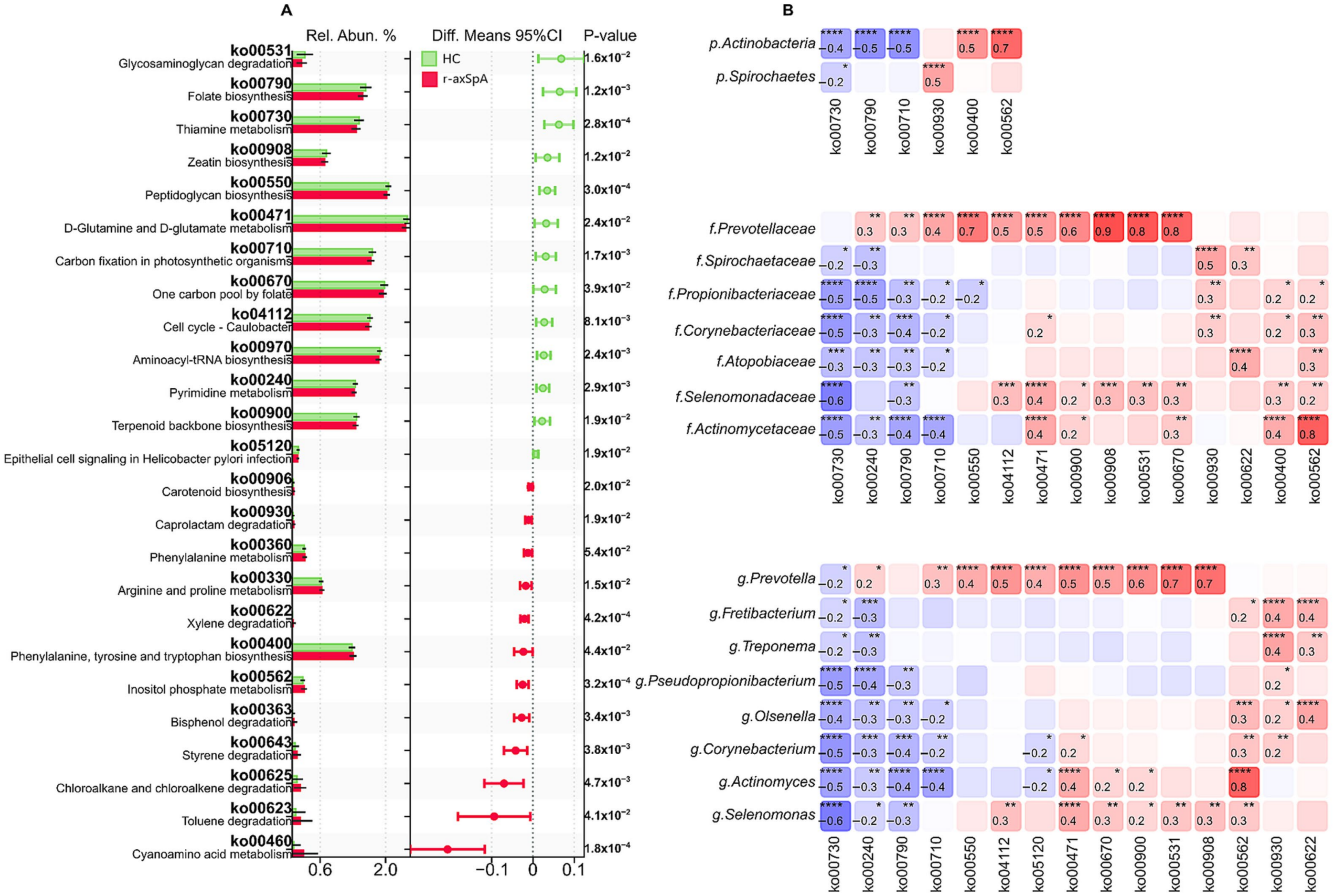
Variation in microbiome functional profiles between healthy controls (HC) and patients with radiographic axial spondyloarthritis. **(A)** Confidence plot of differences between PICRUSt2-predicted pathways abundances. Barplots show mean pathway relative abundance %. Error bars represent 95% confidence intervals (CI) for difference between means; non-overlapping CIs & *p* ≤ 0.05. **(B)** Heatmap of correlations between marker taxa and marker pathways. Spearman’s rho. *p ≤ 0.05; **p ≤ 0.01; *p ≤ 0.001; **p ≤ 0.0001.

To explore the potential functional implications of the oral microbiome in r-axSpA, we performed metagenome functional prediction using the PICRUSt2 algorithm, which infers microbial community functions based on 16S rRNA gene marker sequences. Differential analysis revealed 25 KEGG functional modules that differed significantly between Cntrl and r-axSpA patients. Among these, 12 modules were significantly enriched in the r-axSpA group, while 13 modules were reduced, indicating alterations in the predicted metabolic and signaling capabilities of the oral microbiome associated with the disease state (Figure 5A).

The predicted functional profile of the oral microbiome in r-axSpA patients revealed increased activity in multiple biosynthetic pathways, including carotenoid biosynthesis (ko00906), phenylalanine, tyrosine, and tryptophan biosynthesis (ko00400), and inositol phosphate metabolism (ko00562). In addition, several xenobiotic degradation pathways were upregulated, such as caprolactam (ko00930), xylene (ko00622), bisphenol (ko00363), styrene (ko00643), chloroalkane and chloroalkene (ko00625), and toluene (ko00623) degradation. Enrichment was also observed in amino acid metabolism pathways, including phenylalanine metabolism, arginine and proline metabolism, and cyanoamino acid metabolism.

In contrast, r-axSpA patients exhibited reduced activity in a range of core microbial functions, including glycosaminoglycan degradation (ko00531) and biosynthetic pathways such as folate biosynthesis (ko00790), thiamine metabolism (ko00730), peptidoglycan biosynthesis (ko00550), and terpenoid backbone biosynthesis (ko00900). Downregulation was also evident in D-glutamine and D-glutamate metabolism (ko00471), one-carbon metabolism via folate (ko00670), and cell cycle–associated pathways, including Caulobacter cell cycle regulation (ko04112) and aminoacyl-tRNA biosynthesis (ko00970). Furthermore, pyrimidine metabolism (ko00240), a critical pathway in nucleotide synthesis, was also reduced.

To further link microbial taxa with predicted functional shifts, we performed correlation analyses between marker taxa and marker pathway (Figure 5B) Actinobacteria, significantly enriched in r-axSpA patients, showed strong positive correlations (*r* = 0.7, *p* ≤ 0.0001) with inositol phosphate metabolism (ko00562), a pathway involved in cell signaling, proliferation, apoptosis, and intercellular communication. This functional enrichment, coupled with Actinobacteria dominance, may suggest a dysbiotic immunomodulatory role of these taxa in r-axSpA pathogenesis.

Conversely, Prevotellaceae, notably enriched in healthy controls, exhibited robust positive correlations (*r* = 0.7–0.8, *p* ≤ 0.0001) with key homeostatic pathways, including peptidoglycan biosynthesis (ko00550), folate metabolism (ko00670), glycosaminoglycan degradation (ko00531). These pathways are associated with cell wall integrity, nucleotide biosynthesis, extracellular matrix turnover, and signaling mechanisms, collectively indicating a potential role of Prevotellaceae in maintaining mucosal stability and microbial community homeostasis.

### 3.6 Immunological profile of the studied group

**Figure 6:**
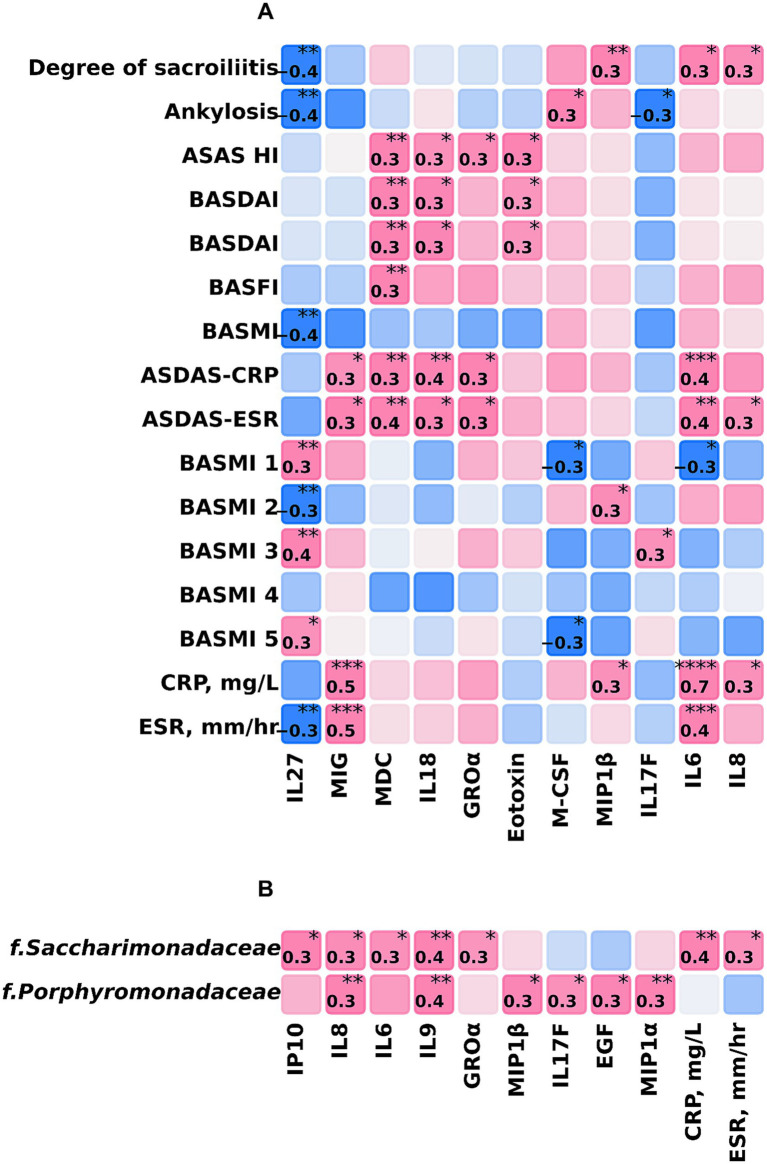
Correlation analysis between the immunological profile, clinical metrics, r-axSpA functional indexes, and marker taxa. **(A)** Heatmap of correlations between immunological profile and r-axSpA clinical metrics/functional indexes. **(B)** Between immunological profile and marker taxa. Spearman’s *ρ*, unadjusted *p*-value. The color scale represents the magnitude of the correlation. Red highlights indicate a positive correlation; blue highlights indicate a negative correlation, all significant correlations are indicated. * ≤ 0.05; ** ≤ 0.01; *** ≤ 0.001; **** ≤ 0.0001.

We conducted a correlation analysis to examine the relationship between the immunological profile, clinical metrics, r-axSpA functional indexes, and marker taxa. The findings revealed several significant correlations (Figures 6A,B).

The ASAS-HI and BASDAI indexes showed positive correlations with MDC/CCL22 and Eotaxin/CCL11 (*r* = 0.3). Broader indexes, namely ASDAS-CRP and ASDAS-ESR, demonstrated additional correlations. These included IL-18, IL-6, and IL-8 (*r* = 0.3–0.4). Both serum CRP and ESR levels correlated with IL-6 and MIG/CXCL9 (*r* = 0.4–0.7). These correlations encompass key proinflammatory cytokines central to r-axSpA pathogenesis.

Examination of correlations between marker taxa *Porphyromonadaceae* (both a marker of r-axSpA, when compared to Ctrl and a marker of inflammation and sacroiliitis within the r-axSpA group) and *Saccharimonadaceae* (taxon of phylum *Patescibacteria*; a marker of inflammation and sacroiliitis within the r-axSpA group) further identified associations with serum cytokines in r-axSpA patients (Figure 6B). Among these, the correlations included a positive association between *Porphyromonadaceae* and IL-8/CXCL8, IL-9, IL-17F, EGF, MIP1α/CCL3, and between *Saccharimonadaceae* and IP-10, IL-6, and IL-9 (*r* = 0.3). These findings indicate that increased abundance of these taxa may be associated with elevated concentrations of key r-axSpA-associated proinflammatory cytokines, although the direction and mechanisms of this association require further investigation.

It should be further noted that while we conducted correlation analysis only for marker taxa using rank correlation, the immunological data demonstrated high dispersion during the analysis, suggesting a greater degree of heterogeneity. Therefore, correlations detected in this analysis may not robustly generalize to other populations, prompting further microbial-immunological research with a larger sample size. Importantly, these correlation analyses were performed without adjustment for multiple testing and are therefore presented as exploratory and hypothesis-generating rather than confirmatory; the resulting associations should be interpreted with caution and require replication in independent cohorts.

### 3.7 Cytokine and oral microbiome signatures as predictors of disease progression in radiographic axial spondyloarthritis

To evaluate the predictive capacity of immunological and microbial features in ankylosing spondylitis, we applied multivariate machine learning models to forecast the presence of structural complications and disease activity. Among all modeled outcomes, the prediction of ankylosis (class ratio 28:29) and spinal kyphosis (class ratio 33:24) showed the highest performance when either circulating cytokines or the taxonomic composition of the oral microbiota were used as input variables.

**Figure 7:**
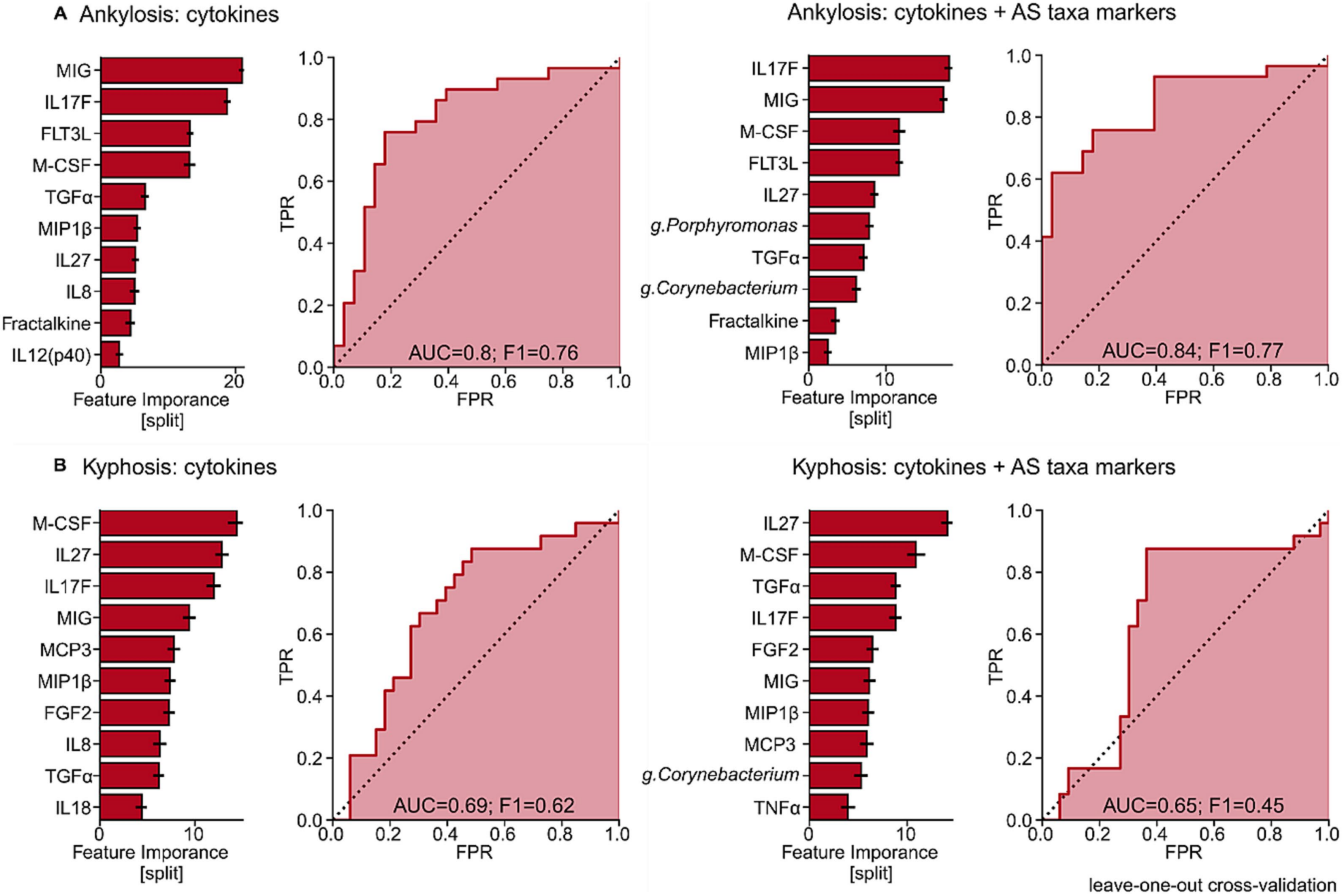
Feature importance and ROC curve for predicting complications in patients with radiographic axial spondyloarthritis (r-axSpA). **(A)** The ROC curve illustrates the predictive accuracy for ankylosis and kyphosis based on the relative importance of cytokine features within the immunological profile of the model. **(B)** The ROC curve illustrates the predictive accuracy for ankylosis and kyphosis based on the relative importance of taxonomic features within the microbial profile of the model.

All statistically significant models, including feature importance scores and ROC curves, are presented in Figure 7.

The model for ankylosis demonstrated the highest performance. Using serum cytokine levels alone, the model achieved good discriminatory power (AUC = 0.80, F1 score = 0.76), with top contributing features being MIG, IL-17F, FLT3L, TGF-β, MIP-1β, and IL-27. Addition of LEfSe-identified oral microbial taxa (primarily *Porphyromonas* and Corynebacterium) further improved prediction accuracy (AUC = 0.84, F1 score = 0.77), indicating a complementary value of dysbiotic oral signatures for identifying patients at risk of ankylosis.

In contrast, predictive performance for spinal kyphosis was considerably lower. Cytokine-based modeling yielded only moderate accuracy (AUC = 0.69, F1 score = 0.62). The addition of oral microbial taxa did not improve, but rather slightly decreased model performance (AUC = 0.65, F1 score = 0.45). This limited predictive ability is likely explained by the strong time-dependent nature of kyphosis, which is heavily influenced by patient age and disease duration. As a result, kyphosis appears to be a less suitable outcome for cross-sectional multi-omics prediction compared with ankylosis.

These findings highlight the outcome-specific utility of integrating cytokine and microbial data in ankylosing spondylitis, with substantially greater predictive value for ankylosis than for progressive spinal kyphosis. It should be emphasized, however, that given the modest sample size, the degree of class imbalance, and the absence of external validation, these predictive modeling results should be regarded as proof-of-concept findings rather than a clinically applicable risk-stratification tool. The reported performance metrics are likely optimistic under cross-validation and require confirmation in larger, independent cohorts before any translational application can be considered.

## 4 Discussion

The inflammation associated with bacteria may be linked to the systemic inflammatory response observed in r-axSpA. The most differentially abundant genera enriched in r-axSpA patients compared to controls in our study were *Actinomyces* and *Porphyromonas*. Indeed, enrichment of *Actinomyces* has been reported in oral samples from RA patients (41). Similarly, a general enrichment of Actinobacteria and a seven-fold increase in *Actinomyces* was reported by (19) in the gut of AS patients. The increased abundance of *Actinomyces* is associated with immune pathways through regulating the NF-κB signaling pathway and with the production of pro-inflammatory cytokines observed in r-axSpA.

Inflammation creates an environment with an excess of reactive oxygen species, which are produced by immune responses. The dominance of Actinobacteria, including species like *Actinomyces*, may be associated with the inflammatory environment of r-axSpA, which favors bacteria that thrive under oxidative stress and altered nutrient availability (42, 43). *Actinomyces* spp. have the ability to adhere to the oral tissue and thereby resist cleansing mechanisms. Specifically, *Actinomyces* is associated with increased epithelial permeability through bacterial components in dental plaque, which may challenge mucosal tolerance and enhance immunogenicity (44). *Actinomyces* has also been found to be strongly associated with periodontitis (PD) in patients with axial spondyloarthritis (25) and with periodontitis in general (45).

In contrast, *Porphyromonas* is not typically directly linked to r-axSpA but is specifically associated with periodontal disease (PD). *Porphyromonas gingivalis*, in particular, has been observed in association with r-axSpA through its role in PD, which has a higher prevalence among r-axSpA patients. The prevailing hypothesis suggests that oral bacteria, including *Porphyromonas* species, may be associated with immune dysregulation and chronic inflammation in genetically susceptible individuals. *Porphyromonas gingivalis* possesses specific virulence factors that can promote inflammation, and this local process may be linked to the systemic inflammatory response seen in axSpA. These observations are summarized in the “oral-pathogen etiology hypothesis” (24), which proposes that periodontal pathogens like *Porphyromonas gingivalis* and *Prevotella intermedia* may be associated with the development of r-axSpA in genetically susceptible individuals. Notably, the abundance of Porphyromonadaceae was also increased in the gut of AS patients, suggesting potential ectopic colonization (46).

In our study, *Actinomyces* emerged as a confident marker of r-axSpA, likely reflecting global shifts in the inflammatory status of patients. In contrast, *Porphyromonas* was identified not only as a status-marker but was also associated with disease progression, showing a significant linear association between *Porphyromonas* abundance and the degree of sacroiliitis. Notably, when investigating markers of axSpA progression, we also observed a strong association between the abundance of Patescibacteria, specifically the *Saccharimonadaceae* family, and the degree of axSpA progression. Patescibacteria’s exact role is under investigation but it has been implicated in arthritis. Studies have shown that the abundance of Patescibacteria is altered in the gut microbiome of RA patients. For instance, its abundance has been shown to be linearly associated with RA progression, being depleted in healthy controls, increased in early RA, and enriched in chronic RA (41). Furthermore, Patescibacteria has been found to be increased in the gut of AS patients (47), although no specific functional associations were found.

In this study, beta diversity analyses revealed significant differences in microbial community structure between the control and r-axSpA groups, suggesting a distinct oral microbiome profile in r-axSpA patients that extends beyond an increase in a few specific taxa. We observed a significant enrichment of *Actinomyces* and *Porphyromonas* across all r-axSpA patients and identified a strong association of the latter with disease progression. This points to the formation of a specific “r-axSpA-associated microbiome” characterized by a proinflammatory shift. This shift involves both an increase in potentially harmful taxa, such as *Actinomyces*, and the proliferation of *Porphyromonas*.

These findings suggest a distinct proinflammatory oral microbiome signature associated with r-axSpA, characterized by an increased abundance of taxa that have been linked to proinflammatory effects and potential disruption of barrier functions. The observed positive correlation between Porphyromonadaceae abundance and serum IL-8, IL-9 and IL-17F levels (*p* < 0.05) further suggests that this altered oral microbial community is associated with sustaining systemic Th17-driven inflammation in axSpA.

In our study, alpha diversity estimates were largely similar between the control and r-axSpA groups, although taxonomic richness showed a marginally significant decreasing trend in the r-axSpA group. This contrasts with other studies that have reported reduced alpha diversity in the oral microbiome of AS and related conditions (26, 28). For instance, Stoll et al. observed reduced alpha diversity in juvenile AS (26), and Gill et al. reported reduced salivary alpha diversity in axSpA patients (28). These microbial shifts may be associated with the pro-inflammatory environment in axSpA, which favors the expansion of specific bacteria such as *Actinomycetaceae* and *Porphyromonadaceae*, thereby promoting dysbiosis and a reduction in phylogenetic diversity. The presence of proinflammatory cytokines (e.g., IL-12p70, IL-8) and harmful metabolites (e.g., cadaverine, putrescine) in the saliva of axSpA patients can further exacerbate this dysbiosis, as these compounds correlate with microbial changes and enhance inflammation (27).

While a strong association exists between oral dysbiosis and r-axSpA, the cross-sectional design of this study does not allow us to determine whether these microbial changes precede, accompany, or result from the disease. Collectively, our results highlight the oral microbiome as a promising target for further research and potential therapeutic intervention in axSpA. However, the cross-sectional design of this study precludes definitive conclusions regarding causality. Larger longitudinal studies incorporating shotgun metagenomics, metabolomics, and functional assays are needed to determine whether the identified “r-axSpA-associated oral microbiome” is associated with disease pathogenesis and the degree of its contribution across different genetic risk profiles.

This study has several limitations. First, the sample size was limited due to stringent exclusion criteria, which may affect the generalizability of the findings. Second, we did not account for lifestyle factors such as smoking and diet that could have influenced the observed microbial profiles. Third, all AS patients had a long-standing disease, which is typically associated with a less aggressive disease course. Fourth, the AS cohort was undergoing active treatment, including non-biologic and biologic DMARDs and NSAIDs, while the healthy controls were not, potentially confounding the microbiome comparisons. Additionally, associations with periodontal parameters were not examined in this analysis due to limited sample size and low variability in the periodontal data, constraining the explanatory scope of the analysis. Because periodontal disease is an established major determinant of oral microbiome composition and is known to be more prevalent in spondyloarthritis, periodontal status represents a major potential confounder whose effect on the oral microbiome cannot be fully disentangled from disease-related changes in the present cohort. The microbial-disease associations we report may therefore partly reflect underlying periodontal status rather than r-axSpA per se, and this limitation should be borne in mind when interpreting our findings; future studies in larger cohorts stratified by standardized periodontal indices will be required to separate these contributions. In addition, the correlation analyses between marker taxa, clinical parameters, and circulating cytokines were performed without correction for multiple testing and are consequently framed throughout as exploratory and hypothesis-generating rather than confirmatory. Likewise, the machine-learning models linking cytokine and microbial signatures to structural outcomes are presented as proof-of-concept findings, given the modest sample size and the absence of external validation, and will require replication in larger, independent cohorts before any clinical or translational application can be considered. Finally, reliance on 16S rRNA gene sequencing limited the resolution and certainty of our taxonomic assignments and precluded direct assessment of the community’s functional potential.

## Data Availability

The datasets presented in this study can be found in online repositories. The names of the repository/ repositories and accession number(s) can be found at: https://www.ncbi.nlm.nih.gov/; PRJNA1358013.
