# Supplementary figures and images for: Oral microbiota alterations in radiographic axial spondyloarthritis
Source: Front Med (Lausanne). 2026 May 12;13:1815404. doi: 10.3389/fmed.2026.1815404 (PMC13201126; doi:10.3389/fmed.2026.1815404)

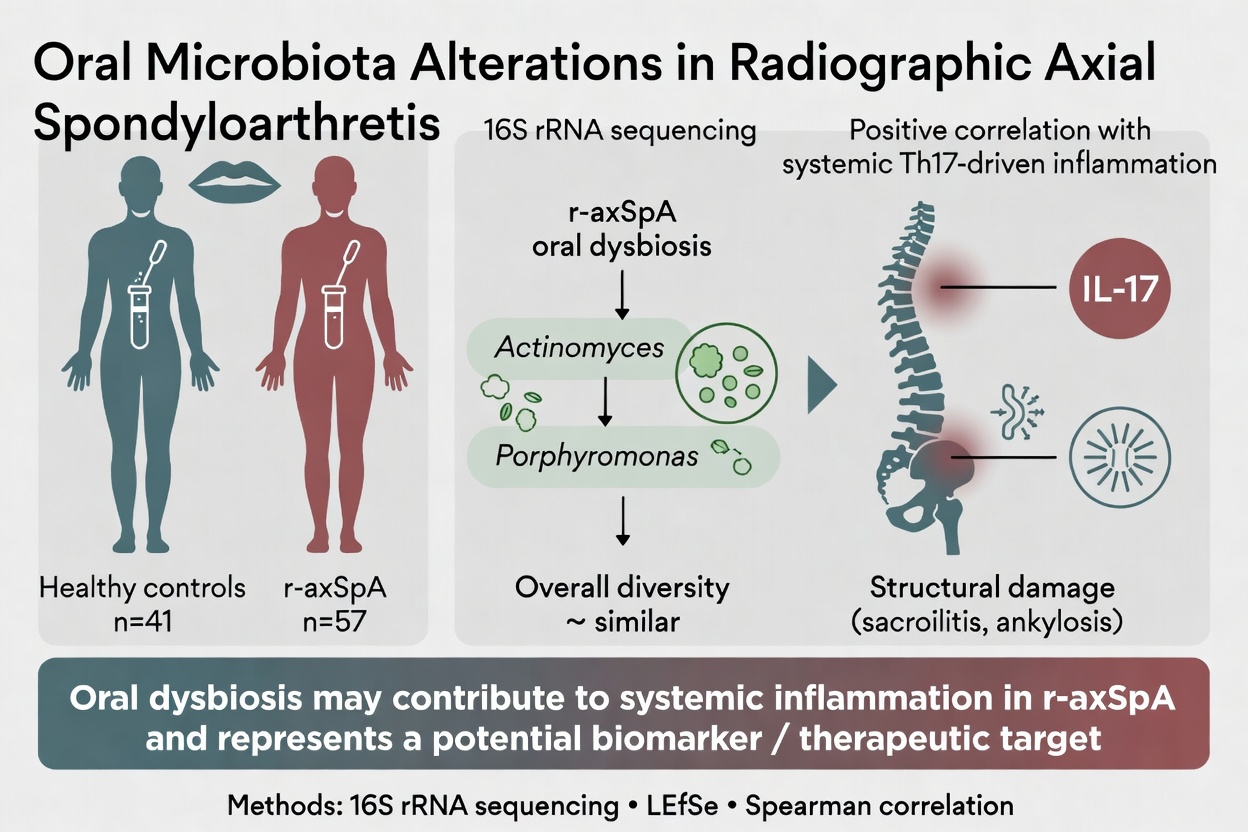

Supplement: Supplementary file 1 [file Image_1.jpeg]
